# Understanding the Role of Controlled Environments for Producing Mycelium‐bound Composites: Advancing Circular Practices for Integrating Biotechnology into the Construction Industry

**DOI:** 10.1002/gch2.202300197

**Published:** 2024-05-30

**Authors:** Tiziano Derme, Francis W. M. R. Schwarze, Benjamin Dillenburger

**Affiliations:** ^1^ ETH Zurich ITA‐Institut für Technologie in der Architektur Stefano‐Franscini Platz 1 Zurich CH‐8093 Switzerland; ^2^ Empa Lerchenfeldstrasse 5 St. Gallen CH‐9014 Switzerland

**Keywords:** circular economy, controlled environments, fungal‐based materials, prefabrication

## Abstract

The architecture, engineering, and construction industry is undergoing a significant shift, steering buildings away from resource‐intensive processes toward becoming instruments for climate mitigation. In this transformative landscape, integrating circular bio‐based alternatives and reducing emissions through biotechnological and enzymatic processes have significant potential. Specifically, mycelium‐bound composites have emerged as renewable alternatives for new materials and added‐value wood products. Despite their numerous advantages, integrating these materials into current engineering practices presents challenges deriving from the complex nature of the material´s production process and the transfer from the laboratory to the industrial scale. In this regard, the design and engineering of novel controlled environments are fundamental in maintaining optimal growth conditions during material production. This, in turn, influences the overall material performance and potential use in construction.

## Graphical abstract

This perspective article examines the role of controlled environments (CEns) in producing mycelium‐bound composites (MBCs). It offers a novel perspective on how the design and characterization of CEns can facilitate the integration of these materials within the construction sector. The successful adoption of such materials offers a blueprint for novel circular models that valorize biological resources and expand the range of applications for MBCs in the construction industry.

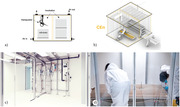

## 1 Introduction

The architecture, engineering, and construction (AEC) industry is facing fundamental challenges that aim to transform buildings from resource‐intensive processes to means for climate mitigation.^[^ ^1^ ^]^ In this context, climate mitigation strategies aim to reduce carbon dioxide (CO_2_) emissions from the primary sources of energy consumption of a building.^[^ ^2^ ^]^ These strategies involve progressive substitutions, replacing fossil heating systems and materials used for thermal insulation with bio‐based alternatives derived either partially or entirely from biomass. Such biomass includes terrestrial and marine plants, their components, biogenic residues, and waste.^[^ ^3^ ^]^ Advancements in biogenic material research have become one of the main focuses in different fields, including biotechnology, bio‐processing, engineering, and construction.^[^ ^4^, ^5^, ^6^, ^7^ ^]^ These domains leverage biogenic materials employing a diverse range of manufacturing techniques that span from extraction and rudimentary mechanical processing of natural fibers to fermentation and advanced enzymatic or catalytic conversions.^[^ ^8^ ^]^ The increasing interest and development of biotechnological applications to the built environment, alongside corporate and academic research, signal a novel material transition. This transition holds the potential to bridge the gap between anthropogenic waste (e.g., chemical or biological waste used as by‐products) generated by industrial processes and the delicate balance of natural environmental cycles.^[^ ^9^, ^10^ ^]^ Bacteria and fungi, in this regard, have gained significant attention as potential agents for creating a wide range of products ranging from microbial cementitious materials^[^ ^11^, ^12^ ^]^to microbial polysaccharides as admixtures for cement and biodegradable bioplastics.^[^ ^13^ ^]^ These microbiological‐driven materials are renewable as they do not rely on specific extractive processes, do not require large quantities of energy for their production, and allow for transformation into other forms, such as nutrients.^[^ ^14^ ^]^ Despite numerous advantages, their integration into current engineering practices is still in its early stages.

### 1.1 Biotechnologies and the AEC Industry

**Figure 1:**
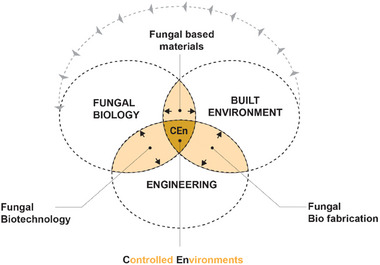
Venn diagram illustrating correlations between CEns, fungal biology, engineering, and the built environment. The intersections give rise to distinct subdisciplines, namely fungal biotechnology, and bio‐fabrication, used to cultivate fungi and manufacture fungal materials. Controlled environments are a pivotal common factor across all disciplines and subdisciplines.

While civil and environmental engineering is conspicuously developing various solutions based on the fundamental know‐how of microbiology,^[^ ^15^ ^]^ this knowledge is often a general application of biological science rather than firmly interlaced and set within design and engineering problems.^[^ ^16^ ^]^ Microbial and fungal–biotechnological processes, similar to other industrial fermentation processes such as cheese and beer production, are characterized by complex variables. These variables include long production cycles, the risk of contamination, and multi‐step manufacturing processes requiring highly specialized knowledge and infrastructure.^[^ ^17^ ^]^ In this context, one of the most significant challenges in scaling up these processes lies in converting laboratory‐scale protocols and controlled environments into industrial‐sized equivalents.^[^ ^16^ ^]^ These environments play a fundamental role, allowing us to conduct operations safely. The limitations imposed by infrastructure and manufacturing constraints directly impact the performance, scalability, and integration into the AEC industry. Simultaneously, the current practices of digitalization and automation introduced by the AEC industry due to the inherent character of the sector of adapting to changes present difficulties in incorporating fundamental advances in biotechnology.^[^ ^18^ ^]^ When contextualized within a specific industry like AEC, the challenges posed by biotechnologies provide an opportunity to re‐think industrial manufacturing, prefabrication models, and material production on a larger scale. Integrating biotechnology into the AEC industry represents a key challenge for introducing and applying new sustainable and circular materials at an architectural scale. In this regard, adapting and characterizing controlled environments (CEns) into specialized manufacturing room scale units engineered for such novel material systems are crucial for enabling scalability, consistency, and expansion of the design possibilities and their future applications (**Figure** **1**).

This perspective article examines the role of CEns and bio‐based materials derived and harvested using wood decay fungi. Specifically, it highlights how the design and characterization of operational features of such specialized infrastructure enable the creation of a new technological framework for manufacturing fungal‐based composites. Mycelium‐bound composites (MBCs) are a product of a solid‐state‐fermentation (SFF) process characterized by the interactions between filamentous fungi, lignocellulosic substrates, and specific environmental conditions.^[^ ^19^ ^]^ The dependency between the environmental conditions, the characterization of the SFF, and the final properties of fungal‐based materials are the critical factors for the low utilization of this material in the AEC industry.^[^ ^20^ ^]^ Within the article, we first outline the essential components of the material formation process, followed by exploring how controlled environments contribute to the final material performance and current industrialization strategies. Furthermore, we offer a fresh viewpoint that illustrates how the characterization of CEns might help with meter‐scale architectural element prefabrication. Here, we address the challenges and opportunities of integrating MBCs into existing construction practices, including scale, design, and knowledge transfer considerations.

The objectives of this article can be summarized as follows:

1. Investigate current research trends on MBCs with a specific focus on SSF and operational variables of CEns.
2. Review current process conditions and industrialization aspects for manufacturing MBCs
3. Identify criteria to determine the impact of CEns on the performance of MBCs
4. Propose novel typologies of CEns to enhance the integration of MBCs into current practices for prefabrication and off‐site construction.
5. To date, the potential for in situ fabrication of MBCs has not been reviewed
6. Provide a qualitative comparison between CEns used in industry and controlled environments that are specialized for the prefabrication of construction elements.

By addressing the above aspects, this perspective article aims to shed light on the significance of CEns for producing fungal‐based materials, their potential for architectural applications, and their comparison to existing industrial processes. Through this study, we find prospects for innovation and enhanced sustainability in the construction sector while contributing to the field's knowledge and understanding.

## 2 Background

In the background of this paper, we dissect three critical areas: fungal biotechnologies for value‐added wood products, fungal bio‐welding, and fundamental industrialization aspects. Specifically, in the first area, we explore how fungal biotechnology transforms wood products by repurposing agricultural and forestry by‐products into high‐value metabolites and engineered wood products. In the second, we delve into fungal bio‐welding, investigating enzymatic fermentation processes for renewable lignocellulose bio‐composites and examining the intricate dynamics of mycelial colonization and resulting material properties. Finally, the third refers to industrialization. It highlights the challenges and opportunities in scaling up fungal‐based material production, covering factors such as process conditions, selection of fungal strains, and the design and operation of production facilities.

### 2.1 Fungal Biotechnology Research for Value‐Added Wood Products

Fungal biotechnological research, in particular, is used to upcycle agricultural and forestry by‐products and waste or to produce high‐value metabolites and engineered and value‐added wood products.^[^ ^21^, ^22^ ^]^ The use of wood decay fungi for biotechnological applications has been studied for decades due to their enzymatic actions and compatibility with the environmental conditions required for wood engineering applications. Knowledge of the wood structure and lignin composition of different cell types in wood enables the interpretation of degradation modes and prediction of whether the wood of a specific tree will be resistant or susceptible to a particular type of decay.^[^ ^23^ ^]^ Depending on the lignin composition, colonization, and weight losses in wood by decay fungi vary significantly. In wood with a higher ratio of syringyl lignin (e.g., beech and sycamore), weight losses by white rot fungi are often considerably higher than in the guaiacyl richer wood of conifers.^[^ ^23^ ^]^ A high adaptation of a wood decay fungus to the substrate helps to counteract contaminations and to produce fungal‐based products in a shorter time, thereby saving energy and resources. Bio‐pulping,^[^ ^24^ ^]^ bio‐bleaching,^[^ ^25^ ^]^ spalting,^[^ ^26^ ^]^ bio‐incising,^[^ ^27^ ^]^ bio‐remediation,^[^ ^28^, ^29^ ^]^ and bio‐welding^[^ ^30^, ^31^, ^32^ ^]^ are some of the most relevant enzymatic processes that have been successfully integrated into various industries.

### 2.2 Fungal Bio‐Welding

Fungal bio‐welding, in particular, holds promise as an enzymatic fermentation process for producing renewable lignocellulose bio‐composites. During the material formation process, the fungal mycelia colonize the lignocellulose biomass, creating a continuous and 3D network that mechanically binds the biomass substrate particles.^[^ ^33^ ^]^ The characteristics and intensity of this colonization/decay process depend on complex dynamics that modify the chemical and physical nature of the biomass, enabling the creation of a wide range of composites suitable for different applications. One of the critical advantages of fungal‐based composites, making them an attractive complementary bio‐based solution for the AEC industry, lies in their inherent renewable nature and versatility in growing on various lignocellulose waste substrates derived from the agricultural or wood industry.^[^ ^34^ ^]^ In the literature and companies commercializing fungal‐based products, we find two main typologies of composites: Pure mycelium materials (PMMs) and MBCs. The former relates to composites formed solely by mycelial mass and generally manufactured using a liquid‐state fermentation process (LSF) or Solid‐state surface formation (SSSF).^[^ ^35^, ^36^, ^37^, ^38^ ^]^ In contrast, the latter is characterized by the growth of a fungal strain on a specific lignocellulose substrate^[^ ^39^, ^40^ ^]^ and is characterized by a SSF process.^[^ ^41^ ^]^ In both methods, the air contained within the mycelial network or between the loosely packed substrates and mycelia matrix results in a low‐density material with foam‐like properties (with a density between 60–300 kg m^−3^) similar to polystyrene and polyurethane.^[^ ^39^, ^42^ ^]^ It can also acquire characteristics similar to those of polymers or elastomers found in natural materials, depending on the kind of characterization and post‐processing.^[^ ^43^, ^44^ ^]^ The performance of these materials, as described by several authors,^[^ ^39^, ^45^, ^46^, ^47^ ^]^ varies depending on the fungal strain selected for the inoculation, the source, type, nutritional profile of the substrate, and environmental growth conditions.^[^ ^43^ ^]^

Post‐processing ultimately influences the material performance more than all the other factors affecting material growth. Cold and heat pressing result respectively in a 2–43 fold density increase with an improvement in homogeneity, rigidity, and tensile strength that improve the range of the performance of the material from being foam‐like^[^ ^48^, ^49^, ^50^ ^]^ (0.6–2 MPa elastic modulus given a range of densities for both tension and compression) to natural like^[^ ^51^, ^52^ ^]^ (2.2–4.4 Mpa of compressive strength). Additionally, MBCs exhibit remarkable properties such as biodegradability,^[^ ^53^ ^]^ resistance to flammability,^[^ ^34^ ^]^ acoustic absorption (70–75% reduction of frequencies > 1500 Hz with a Noise‐Reduction‐Coefficient ranging from 0.4–0.53),^[^ ^54^ ^]^ and thermal conductivity (0.04–0.08 W mK^−1^),^[^ ^55^, ^56^ ^]^ similar to traditional insulation materials.

Companies commercializing MBC´s materials in the AEC industry favor using the material for interior and non‐structural applications. These applications typically produce planar elements with variable dimensions and thicknesses ranging from 50 to 100 mm, making them suitable for producing modular assemblies and interior fit‐outs. Current uses include acoustic paneling, desk dividers, insulation boards, cores for interior partitions, drywalls,^[^ ^57^, ^58^ ^]^ and substitutes for materials like medium‐density fiberboards (MDF), oriented strand boards (OSB), or plywood.^[^ ^57^ ^]^ Despite their promising properties and applications, the adoption and scale of MBCs in the AEC industry still need to be improved compared to other construction materials. This disparity stems from numerous factors, as extensively discussed in previous articles and reviews.^[^ ^20^, ^34^, ^50^, ^58^ ^]^ First, MBCs, like other bio‐based and wood‐based products, exhibit limitations in mechanical performance, needing more strength and stiffness for load‐bearing applications. Moreover, their susceptibility to wear, abrasion, dynamic impacts, and poor resistance to sanding, cutting, scratching, and general wear present challenges.^[^ ^59^ ^]^ Enhancing usability often entails lamination with other materials or coatings, particularly biodegradable polymers like PLA or PHA, especially for applications in warm and humid environments.^[^ ^60^ ^]^ Additionally, durability concerns arise due to sensitivity to weathering and moisture uptake, attributed to colonization by molds and bacteria.^[^ ^51^ ^]^ Despite prevalent lab testing, limited field tests under various conditions highlight these vulnerabilities. Furthermore, the complexity of the manufacturing process poses scalability challenges. Unlike traditional construction materials such as steel, concrete, bricks, and wood, the production of MBCs involves a complex multi‐step process requiring highly specialized operators. Consequently, these conditions impact the competitiveness of MBCs in terms of costs, availability, and production time compared to conventional materials.^[^ ^61^ ^]^

Specifically, we find that most of the published and accessible data concerning the limitations of MBCs as construction materials reveal a disparity between their performance for specific use‐cases and their compliance with the European Technical Approval Guidelines (ETAGs),^[^ ^62^ ^]^ and the relevant Eurocodes available for bio‐based building products.^[^ ^63^ ^]^ This misalignment poses challenges particularly in comparing MBCs with other construction materials whose performance and standard basic requirements are determined by the Construction‐products regulations (CPR)^[^ ^64^ ^]^ and European Conformity (CE) marking protocols (**Table** **1**).

**Table 1:** Summary of the basic work requirements (BWR) for the harmonized standard EN13986 for an equivalent material product such as Wood‐based panels for use in construction.

Basic work requirement (BWR)	Key parameters^a)^	Application	Performance
1	Mechanical resistance and stability	Structural, Non‐Structural	strength/stiffness
2	Safety in case of fire	Structural, Non‐Structural	fire resistance
3	Hygiene, Health and Environment	Structural, Non‐Structural	chemical, biological impact
4	Safety and Accessibility in Use	Structural – all conditions	durability, impact resistance
5	Protection against noise	Structural, Non‐Structural	sound insulation, absorption
6	Energy, Economy, and Heat Retention	Structural, Non‐Structural	thermal conductivity
7	Sustainable Use of Natural Resources	Basic works	material composition/origin

^a)^ Regulatory reference‐based the harmonized standard EN 13 986:2004 Wood‐based panels for use in construction.^[^ ^65^ ^]^ Characteristics, evaluation of conformity and marking.

#### 2.2.1 Process Conditions for Solid‐State Fermentation of Fungal‐Based Materials

**Figure 2:**
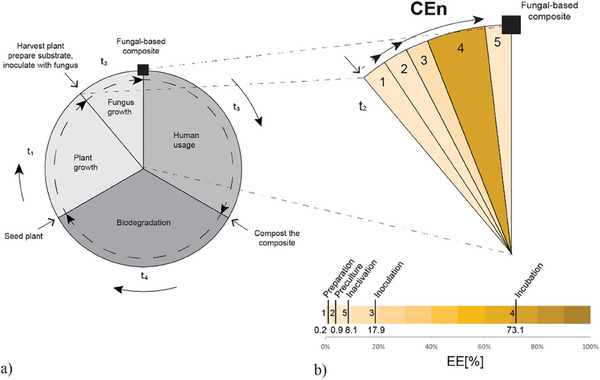
a,b) Temporal cycle of MBCs. Adapted graphics and reproduced under terms of the Creative Commons CC‐BY license.^[^ ^69^ ^]^ 2022, Springer Nature. Primary temporal frame t1) growth time of natural fiber, t2) Following, material preparation, inoculation, and initiation of growth, followed by harvesting and processing of the fungal‐based material, t3) lifespan of material usage, t5) end of life and material biodegradation; b) Definition of the energy impact of fundamental phases of material formation. The presence of a controlled environment in steps 3 and 4 illustrates the most critical phases and highly specialized operational conditions. Data adapted from available published resources.^[^ ^66^, ^68^ ^]^

Within the processing of MBCs, the intricate interplay between process conditions and solid‐state fermentation (SSF) profoundly shapes the resultant material properties and the aforementioned limiting factors.^[^ ^66^ ^]^ Notably, the process for producing a standard “mycoblock” brick‐like material consists of five essential steps: 1) preparation of fungal cultures and grain spawn, 2) preculture, 3) inoculation and pre‐growth, 4) incubation and transfer into a mold, 5) Inactivation and processing (**Figure** **2**).^[^ ^67^ ^]^ Depending on the fungal species and biomass production, the cultivation conditions and incubation periods may change for every step. The incubation period in steps 3 and 4 is usually the most incidental phase during mycelial growth and stands out as the most energy‐intensive.^[^ ^66^, ^68^ ^]^

The incidence of the incubation phases is directly related to the growth performance within the SSF process, CEn, or bioreactor. Generally, SSF is particularly advantageous for incubating fungal‐based materials and MBCs as it resembles natural growth conditions.^[^ ^70^ ^]^ The cultivation of the material employs a solid substrate with adequate moisture content for the maintenance, growth, and metabolic function of the fungal species. Identifying the optimal environmental conditions for the enzymatic action and the consequent material formation requires the control of parameters such as temperature, moisture content, and air‐flow rate.^[^ ^71^ ^]^ These should be considered separately, but also through the interactions between each other. Such environmental variables coincide with the operational functions of a CEn. The impact of temperature for various fungal strains cultivated during SSF has been documented chiefly for finding optimal values for enzyme production and only marginally for the output of MBCs.^[^ ^71^, ^72^, ^73^ ^]^ This characterization approach can serve as a valuable tool for anticipating the consequences of temperature variations of SSF, bioreactors, and, subsequently, fungal‐based materials. The ideal moisture content for the production of MBCs in an SSF process depends simultaneously on the lignocellulosic substrate and the applied fungal strain.^[^ ^74^, ^75^ ^]^ A critical consideration is that the initial moisture content varies during incubation and the material formation period. In addition to regulating moisture content and temperature, air exchange plays a fundamental role in producing fungal‐based materials. Within an SSF, the function of aeration is to maintain aerobic conditions, lower the access of CO_2_ generated from the fungal growth, and regulate the moisture and temperature content of the substrate during incubation.^[^ ^76^ ^]^ The balance between environmental variables paired with selecting suitable lignocellulosic substrates defines and stimulates a precise degradation pattern that differs among different wood decay fungi.^[^ ^77^ ^]^ For example, environmental fluctuations within an SSF can trigger decay fungi to switch to simultaneous or selective delignification types of white rot and vice versa.^[^ ^78^ ^]^ The degradation of hemicellulose, cellulose, or lignin has a direct impact on the final material properties and its potential use (e.g., white rot fungi preferably degrade lignin and tend to be used for the production of low‐density materials or materials where mechanical strength is not a priority).

The scalability of SSF processes on an industrial scale, i.e., enzyme production, has been impeded primarily by challenges associated with monitoring and controlling various process variables.^[^ ^79^, ^80^ ^]^ Similarly, in the AEC industry, the significance and utilization of SSF and CEn in the development of MBCs are frequently underestimated and inadequately considered. This underestimation is a key obstacle hindering the widespread application of fungal‐based materials in the AEC industry.

### 2.3 Industrialization Aspects

The industrialization process for large‐scale production of fungal‐based materials presents similar requirements and challenges typical for all SSF processes used to produce enzymatic cellulase products from filamentous fungi. Cellulase production during SSF processes has characterized most scientific publications over the last ten years, for example, regarding bioethanol production from lignocellulosic biomass.^[^ ^81^, ^82^ ^]^ The fundamental aspects of applying SSF for MBCs are the design of the production process, operation, and scalability. There is a direct relationship between the operation conditions (e.g., temperature, aeration, moisture content, composition of the substrate) and the yield of fungal growth.^[^ ^70^ ^]^ Fluctuations or adverse humidity and temperature gradients may negatively affect the process's scalability and the material's performance, hampering its viability and commercialization.^[^ ^74^ ^]^ It is, therefore, essential to understand the features of the SSF processes and operational methods for applying MBCs in the AEC industry (**Table** **2**).

**Table 2:** Essential physical and operational variables, their effect, and the overall range of variability are to be considered when planning a controlled environment for producing MBCs.

Type	Variable	Effect	Range of variability^g^ ^)^
Physical	Design	cost, customization, and automation	High^a)^
	Volume (m^3^)	Scalability, cost, customization, and automation	High^b)^
Operational	Relative Humidity (%)	growth performance and durability	60–100^c)^
	Temperature (°C)	growth performance and durability	25–32 ^d)^
	Carbon Dioxide (ppm)	growth performance and durability	400–5000 ^e)^
	Fresh Air exchange (m^3^/s)	growth performance and durability	High ^f)^

^a)^ Depending on the type, workability of the substrates and fungal species used;

^b)^ Reliant on the expected volumetric capacity of the facility/unit;

^c–e)^ Subject to the specific requirements of the fungal species used;

^f)^ Depending on the desired CO_2_value;

^g)^ Average ranges derived from data of companies commercializing mushrooms based products.^[^ ^83^ ^]^

In this paragraph, we review SSF processes used by commercial companies, highlighting common and differentiating factors in design operational strategies. Current challenges in the industrialization of SSF applied to the production of MBCs are also discussed.

**Figure 3:**
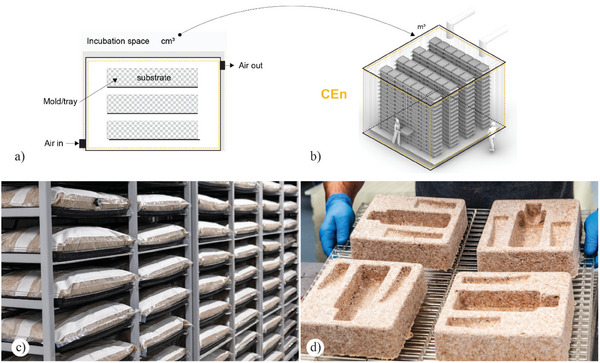
a–d) The conceptual illustration describes a facility for producing MBCs: the dashed line symbolizes the volume of the controlled environment. a) Diagrammatic representation of a standard tray‐bioreactor. Adapted graphics and reproduced under terms of the CC‐BY license.^[^ ^86^ ^]^ b) Volumetric image of current strategies for scaling up the production of fungal‐based materials using trays‐bioreactors. c) Interior view of the facilities used by Ecovative^[^ ^87^ ^]^ and the shelf systems to store effectively the spawn during the pre‐growth phase. d) Low‐density foam material resulting from the harvesting and drying process. c,d) Reproduced with permission by Ecovative.^[^ ^88^ ^]^ Copyright 2023, Ecovative.

From an operational perspective, companies commercializing fungal‐based material products use large trays, bioreactors, or CEns.^[^ ^84^ ^]^ Despite their limitations, due to large operational areas, contamination control, and the difficulty of fully automating the SSF process, they are currently the most suitable models for scaling up the production of MBCs.^[^ ^82^ ^]^ Such bioreactors consist of a chamber/or environmentally controlled indoor environments containing individual trays generally made of thermoplastic materials that can be easily sterilized and customized by shape and specific volumetric requirements. Such trays include the inoculated substrates in static conditions. Usually, the trays are open on the top, have perforations at the bottom, and are stored vertically, leaving a void space in between to increase oxygenation. Additional aeration may be provided into the bioreactor chamber/space and circulate around the tray with humidity and temperature control. To improve consistency and minimize contamination events during production, substrates are generally first inoculated and grown in small batches and only transferred into molds after a period of pre‐growth (**Figure** **3**). This process uses plastic bags made of gas‐permeable‐liquid‐impermeable material (e.g., polypropylene) instead of rigid trays. Such bags allow the growth in static conditions and respiration of the fungal species while maintaining an optimal moisture content in the substrate. One differentiating factor in the operational function of an SSF oriented on producing fungal‐based materials is active growth versus passive growth. The dynamic growth process of the mycelium results in the introduction of significant levels of CO_2_ within the growth chamber. This approach is based on the established knowledge that an elevated CO_2_ environment promotes mycelium growth while inhibiting the development of fruiting bodies. Publicly available information suggests that companies like Ecovative, with their Forager materials, MycoComposite, and AirMycelium, utilize an active growth process, whereas Mogu and Mycowork, with their Reishi and Fine Mycelium, employ passive growth conditions.^[^ ^85^ ^]^

Despite the advantages of trays bioreactors in terms of scalability and high volumetric production capacity, such technologies have specific limitations. These refer specifically to mass and heat transfer limitations in tray bioreactors, which may result in significant internal temperature gradients and elevated gas concentrations when the substrate depth exceeds 40 mm.^[^ ^86^ ^]^ It has been noticed, for example, that within a tray of 50 mm depth cultivated with *Rhizopus oligosporus* at 37 °C with rice bran, the internal temperature of the substrate can rise up to 50 °C, which can have a lethal effect on the mycelium.^[^ ^88^ ^]^

The emphasis on standardizing SSF and CEns processes, as adopted by companies engaged in the commercialization of fungal‐based materials, tends to promote the integration of these materials into particular markets, such as packaging or the production of planar insulation sheets. However, this approach hinders the diversification of their use in the AEC sector. The manufacturing processes associated with such standardized practices need to align with current automation and digital fabrication advancements. Such advancements consider innovative design to fabrication workflows, the fabrication of highly customized building components, scalable fabrication processes driven by target performance, and reliable strategies for repair and material replacement.^[^ ^89^, ^90^, ^91^ ^]^

#### 2.3.1 The Selection of Fungal Strains

The selection of fungal species plays a critical role in effective biomaterial production and is one key factor for competitiveness and patenting of companies that commercialize fungal‐based materials. Criteria for selecting the species include hyphal density and structure, growth rate, substrate/media cost, specific degradation pattern, cultivation conditions, and competitiveness against microbial contaminants.^[^ ^5^ ^]^ As reported by diverse scientists, fungal species belonging to the phylum *Basidiomycota* may be more suitable for biomaterial production due to their strong vegetative growth and capacity to express high‐level enzymatic lignin degradation.^[^ ^92^ ^]^ The increased occurrence in the claims of granted patents shows that such phylum is an ideal candidate for enzymatic processes related to bioprocessing and bioconversion of lignocellulosic substrates in various industrial settings.^[^ ^93^, ^94^, ^95^ ^]^ Specifically, their inner cellular structure and hyphal systems allow the production of a dense hyphal network favorable for more extensive nutritional transportation.^[^ ^96^ ^]^ Such a condition leads to a more robust and thicker hyphal network and the consequent creation of materials with higher compression strength and increased stiffness properties. Characterizing the SSF operational conditions required for most *basidiomycetes* can slightly change depending on the species used. However, despite minor fluctuations, most conditions can be considered universal for the entire phylum. First, the SSF occurs in the absence of light. Light is an indicator that stimulates the production of fruiting bodies; it slows the growing process of mycelium and should be prevented during biomaterial production. Second, the moisture content in the air chamber needs to be relatively high, up to 90%−100%. It can be reduced depending on the fungal species and the moisture‐retaining properties of the substrate used for mycelial growth. Additionally, the metabolic activity of the hyphae requires oxygen and produces C0_2_. Lower levels of C0_2_ stimulate the production of fruiting bodies. Therefore, C0^2^ levels should be maintained to ensure optimal hyphal development throughout the incubation period. Finally, temperature during the incubation varies depending on the species, but generally, temperatures for all *basidiomycetes* should not exceed 40  °C. The temperature should be considered a variable applied to the entire incubation chamber and measured within the substrate. Most *basidiomycetes* have an optimal growth at 25–32  °C.^[^ ^97^ ^]^ It has been reported extensively that the growth phase is the heat transfer across the substrate, inhibiting mycelial growth.^[^ ^98^ ^]^

High‐quality strains are typically selected to enhance product quality, often through antibiotic treatment or genetic engineering, to induce tolerance to bacterial competition and enable growth in climate‐controlled facilities maintained at room temperature (20–22 °C). Notably, the inactivation of the hydrophobin gene sc3^[^ ^99^ ^]^ forms mycelium material with significantly increased tensile strength, with performances up to three to four times higher than fungal‐based composite.^[^ ^100^ ^]^ This increase is attributed to the enhanced mycelial density.

From an industrial perspective, the vast use of *basidiomycetes* for fungal‐based production allows the standardization of room‐scale tray bioreactors and homogenization of all the required variables during the SSF process. The use of specific phylum and the genetic modification of fungal strains present advantages for industrialization and scaling up fungal‐based materials that can be transferred to other industries, such as the AEC sector. Such strategies may allow diversification in the production by using a diverse range of fungal species within one single climatic range of temperature and relative humidity.

## 3 Perspective

### 3.1 The impact of Controlled Environments for Fungal‐Based Materials

Evaluating the combined influence and impact of SSF and CEns for integrating MBCs in the AEC industry is a multifaceted task. It is intricately related to both the physical and operational constraints of a specific infrastructure as well as the inherent characteristics of the composite. Despite the limited literature addressing this condition, it is possible to outline the primary components driven by the type of SSF, bioreactor, and CEn. These components can be synthesized into six interdependent elements.

#### 3.1.1 Performance and Durability

The formation of a solid and interconnected 3D network of hyphae within the selected substrate is highly dependent on the operational variables of the incubation environment CEn and the SSF process.^[^ ^101^ ^]^ Therefore, ensuring optimal growth conditions is essential in promoting the development of a well‐structured hyphal network and a material with consistent properties such as strength, density, and thermal insulation.^[^ ^102^ ^]^ It has been shown in the literature that with an increase in incubation time, a variation of biomass, density, and presence of chitin in the material is evident. Such variation, in most cases, affects the overall homogeneity of the material and its compressive strength. Like other fermentative processes, the material formation process can be described as a kinetic process in which we find a variation in biomass and substrate content determined by O2 or CO2 production and heat exchange.^[^ ^103^ ^]^ Such a relationship has been described mathematically as dependent on time and temperature and implicates the necessity of a CEn. The possibility of precise control of the operational functions facilitates standardization of the process and reproducibility and consistency of material properties.

#### 3.1.2 Production Scalability

The design of and operational characteristics of the bioreactors in SSF are critical factors affecting the potential scalability and transition of fungal‐based materials from lab‐scale to market implementation. Due to significant considerations, the number of bioreactor types at the pilot scale and in the industry is relatively narrow.^[^ ^86^ ^]^ Heat removal becomes challenging beyond a critical substrate quantity, restricting available design strategies.^[^ ^82^ ^]^ The choice of bioreactor type is influenced by factors such as substrate nature, the need for pretreatment, appropriate inoculation procedure, required sterility levels, acceptable contamination level, handling, ease of filling, emptying, and cleaning the bioreactor or controlled environment.^[^ ^104^ ^]^ The bioreactor design must consider these complex variables to ensure an efficient SSF process. Critical process parameters include aeration, heat, and mass transfer, directly impacting the degree, type, and overall volumetric capacity in the prefabrication process. Tray bioreactors represent a viable and flexible way to scale up and pass from bioreactors considered functional units to rooms scale (CEn) with large volumetric material production and products with different sizes, degrees of customization, and prefabrication.

#### 3.1.3 Integration with Prefabrication Processes

The use of large tray‐bioreactors or room‐scale CEn as a model for the industrialization of MBCs facilitates the integration of innovative digital fabrication technologies beyond molding. There has been an increasing interest in diversifying the manufacturing methodologies applied to fungal‐based materials over the last few years, especially in the academic environment. Amongst the most relevant published examples, we find knitted scaffolds,^[^ ^105^, ^106^ ^]^ direct deposition of inoculated substrates using additive manufacturing (AD),^[^ ^107^ ^]^ the use of filament deposition printing for the production of custom‐made molds,^[^ ^108^ ^]^ woven temporary scaffolds pre‐assembled using robotic arms,^[^ ^109^, ^110^ ^]^ and the use of subtractive manufacturing for the fungal of custom volumetric parts.^[^ ^111^ ^]^ Obvious research gaps appear in the previously mentioned projects. Superficial growth, partial contamination, and lack of exhaustive comparative data show the scarce utilization of adequate infrastructure for conducting experiments at a meter scale. The accessibility to specialized infrastructure such as CEns becomes crucial for extending the prefabrication and utilization of fungal‐based materials.

#### 3.1.4 Cost Considerations

The market and pricing of fungal‐based materials are intricately tied to the performance and attributes of the SSF process, along with the presence of the room‐scale CEns. From the few available companies publishing their pricing, the cost of a fungal‐based insulation panel measuring 1200 × 600 × 60 mm varies between 60–300 EUR sqm^−1^.^[^ ^112^, ^113^ ^]^ According to a recent economic assessment, the production cost of a generic mycelium‐based block (MBB) is 17.93 EUR per cubic meter (m^3^).^[^ ^114^ ^]^ The evident gap between the market and production value is critical, particularly considering the anticipated need to use MBCs over traditional fossil‐based ones. The significant cost differential can derive from multiple factors, including the current low market demand, the cost of royalties for utilizing the *MycoComposite* patent,^[^ ^108^, ^109^ ^]^ and the necessity to use a specialized infrastructure and energy resource‐intensive equipment during inoculation, incubation, and processing. Notably, the operational requirements of a controlled incubation environment substantially affect the overall embodied energy profile of the material.^[^ ^68^ ^]^

#### 3.1.5 Supply Chain and Sourcing

CEn ensures a consistent and reliable supply of mycelium, which is crucial for production. By controlling the indoor growth conditions, the mycelium can be cultivated all year round, reducing dependency on seasonal variations and geographical situations.^[^ ^115^ ^]^ However, the availability of suitable agricultural waste or timber byproducts for mycelium growth may still need to be improved within the supply chain.

#### 3.1.6 Regulatory Hurdles and Standards

The controlled environment can aid in meeting regulatory requirements and building standards, as the resulting mycelium‐based composites can be engineered to meet safety, fire resistance, and structural performance standards. Producing consistent materials in controlled environments helps address regulatory hurdles and facilitates acceptance in the AEC industry. Fungal‐based materials are classified as mushroom and mycelium products (MMP) and are equivalent to food or agricultural products. These products, depending on the fungal species used, may be subject to Regulation (EU) 2015/2283 (The general food law) on novel foods.^[^ ^116^ ^]^ Such regulations are part of policies designed by the European Green Deal to make the Union climate‐neutral by 2050.^[^ ^117^ ^]^ Such a regulatory framework requires the material to be subjected to specifications associated with the food industry. Amongst these requirements, we find the need to specify a) biological source and taxonomic information about the fungus, b) the origin of fungal strains, c) laboratory conditions, d) substrate information, and e) type of cultures.

Overall, the controlled environment is crucial in ensuring mycelium‐based composites' scalability, consistency, and quality, addressing some of the mentioned challenges. It allows for controlled production, improved material performance, and compliance with industry standards, influencing their broader adoption in the AEC industry.

### 3.2 Solid‐State Fermentation as a Novel Manufacturing Process for Prefabrication

Current practices adopted by industries utilizing fungi for enzymatic production and companies' efforts in commercializing MBCs showcase a practical methodology for implementing and automating SSF processes on a construction scale.^[^ ^72^, ^86^, ^118^, ^119^ ^]^ This approach provides an enclosed bioreactor‐like controlled environment (CEn) to optimize material and enzymatic production while maintaining consistency throughout the incubation period.^[^ ^104^, ^120^ ^]^ In particular, the adoption of fungal‐based materials into the construction industry, beyond current material performance limitations, is directly associated with the knowledge transfer of SSF and CEns as complementary processes for manufacturing building components. To effectively introduce MBCs within a new manufacturing framework, it is crucial to consider novel construction innovation techniques carefully, diversify manufacturing scenarios, and ensure that these materials align with the specific regulatory and performance requirements of building structures.^[^ ^2^ ^]^ A potential starting point for such integration is to pair SSF and CEns with current efforts to minimize the uncertainty and complexity of the construction processes with the standardization of building systems and repetition of building modules. Such tendency can be summarized as “Industrialized building system,” “prefabricated buildings,” or “modular construction.”^[^ ^121^, ^122^ ^]^ Off‐site fabrication, prefabrication, pre‐assembly, and modularisation offer an opportunity to integrate SSF and CEn into a broader operational and technical framework in which the production of 2‐D or 3‐D fungal‐based modules is approached through design, manufacturing, logistics, and assembly considerations. Prefabrication encompasses the off‐site production and pre‐assembly of components, which are later transported and assembled on‐site as closed or open systems.^[^ ^123^ ^]^ While the term “prefabrication” can have broad applications in the construction industry, it is often categorized into three main streams: a) non‐volumetric off‐site construction (e.g., elements that do not enclose a usable space), b) volumetric off‐site construction (e.g., pre‐assembled parts that define an enclosed usable space), and c) modular buildings(e.g., complete building or portion of it).^[^ ^18^ ^]^ Most MBCs commercialized in construction primarily produce non‐volumetric parts – planar sheets of low‐ and high‐density materials. This condition is fundamentally due to the current design and operational constraints of SSF and CEns. The design, control, and characterization of the SSF and CEn processes^[^ ^104^ ^]^ may play a crucial role in prefabrication practices and scale‐up with fungal‐based materials beyond non‐volumetric building components.

### 3.3 The Characterization and Design of Controlled Environments

The characterization, engineering, and design of CEns in an SSF process are crucial for extending the utilization of MBCs for the AEC industry. Amongst the benefits such infrastructures may provide, we find 1) the improvement of the volumetric and dimensional capacity of prefabricated fungal‐based material components, 2) an increased and significant degree of customization, 3) minimization of material handling during the SSF process, 4) an increased capacity for monitoring fungal growth during the incubation process. The following strategies are introduced as modifications to Trays‐bioreactors aiming to enhance three pivotal components in accordance with fundamental advances in digital design, manufacturing, and prefabrication.^[^ ^124^ ^]^ These components concern the articulation of geometric complexity, the level of customization, and the seamless integration with automated fabrication processes. Such novel CEns can be summarized as follows: a) Integrated controlled environments (INT‐CEn), b) Standalone controlled environments (STAND‐CEn), c) Controlled environments for bulk wood (BULK‐CEn). On a qualitative level, such a technically controlled space provides a more versatile framework for producing MBCs compared to current practices.

#### 3.3.1 Integrated Controlled Environments (INT‐CEn)

**Figure 4:**
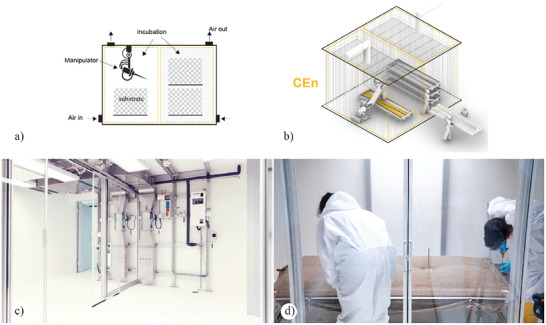
a–d) The conceptual illustration describes the fundamental components of an INT‐ CEn: the dashed line symbolizes the volume of the controlled environment and the potential climatic zone. a) Diagram of the adaption of the bioreactor and fundamental components. b) Volumetric representation of the framework for scale‐up. c) Bio‐Fabrication Lab, ETH Zürich, Switzerland. Reproduced with permission.^[^ ^125^ ^]^ d) Incubation of a significant volumetric prefabricated MBC element.

An Integrated‐controlled‐environment combines clean rooms or specifically engineered spaces that maintain a very low concentration of airborne particulates and tray bioreactors, providing a platform that serves two primary functions. The first concerns the flexibility of operational variables, including temperature, humidity, and air control, along with an increased volumetric capacity for incubating significant architectural components (**Figure** **4**). These environments can accommodate various climatic zones, facilitating simultaneous experimentation with different fungal species or temperature and humidity gradients. The second function, contingent on their volumetric characteristics (measured in cubic meters of available space), offers favorable conditions for integrating innovative manufacturing processes, such as additive manufacturing. Additionally, it can consolidate two crucial stages of material production and the SSF process: 1) inoculation and 2) incubation. Traditionally, these operations occur in separate environments, necessitating the transfer of substrates between phases. In qualitative terms, INT‐CEns facilitate two main prefabrication scenarios: a) automated prefabrication for highly customizable substrates and b) serial fabrication of modular elements with non‐standard substrates. Both scenarios involve the integration of digital manufacturing into the SSF processes, opening up the possibility to utilize a broader range of substrates, including bulk wood, preassembled or pre‐compressed lignocellulosic scaffolds, and specific substrates that require distinct handling conditions. Capitalizing on their volumetric capacity, CEns enable the integration of robotic arms for precise inoculation, automating key phases like inoculation and positioning. This minimizes contamination risks and streamlines handling operations, encompassing the positioning of elements with guided and particular inoculation.

The integration of CEns facilitates various digital manufacturing processes, including rapid prototyping^[^ ^1^ ^]^ and robotically assisted techniques. Currently, the use of these environments is limited due to the required starting expenses of the infrastructure. Experimentation in this direction comes mainly from academic institutions and research groups explicitly focusing on biomaterials.

#### 3.3.2 Stand‐Alone Controlled Environments (STAND‐CEn)

**Figure 5:**
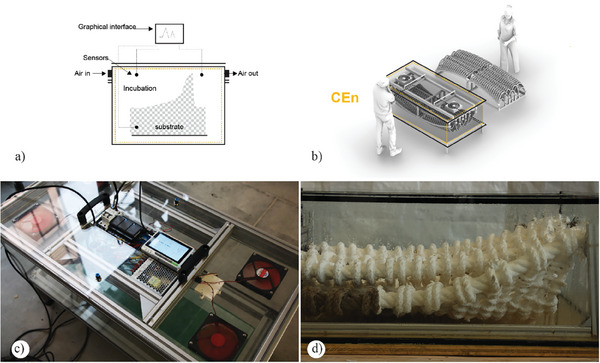
a–d) The conceptual illustration describes the fundamental components of a STAND‐CEn: the dashed line symbolizes the volume of the controlled environment and the potential climatic zone. a) Sectional diagram of the adaption of the bioreactor and its fundamental components; b) volumetric representation of the framework for scale‐up; c) detail of myco‐monitor, created for the production of prefabricated units of 0.5 m^3^; d) prototype resulting after incubation into the stand‐alone controlled environment.

A stand‐alone controlled environment, similar to bioreactors, operates independently from surrounding conditions. These environments are particularly valuable for addressing challenges associated with manufacturing consistency and monitoring in customized processes, where mycelium may lack material uniformity. STAND‐CEn can be adapted, tailored to particular sizes, and tuned to specific environmental control cycles. Specifically, it supports two main actions: a) growth monitoring and data collection of a singular prefabricated element during incubation and b) as an all‐in environment, it allows the integration of the primary SSF sequences without the need for additional infrastructure (**Figure** **5**).

- The first features a network of sensors, a graphical interface, and a control unit that enables extended growth monitoring during the incubation of a mycelium‐based composite architectural component. Estimating fungal growth in SSF processes is challenging due to difficulties separating biomass from the substrate matrix and hyphal penetration into the solid substrate. Thus, a distributed network of sensors facilitates the use of analytical methods for measuring fungal growth. For instance, respirometric analysis has been identified as a viable methodology for biomass measurement during the SSF process.^[^ ^126^ ^]^ This method involves monitoring respiratory gases during an incubation cycle, including consumed oxygen or CO_2_ produced during metabolic activity. Meticulous monitoring results in enhanced data collection, providing more information for predictive material modeling and improving consistency in material properties. It aids in certifying the singular prefabricated unit, ensuring quality control and compliance with regulatory standards such as Regulation (EU) 2015/2283 for novel foods.^[^ ^116^ ^]^
- These environments offer significant advantages by condensing the main fabrication phases into a single functional unit. Operations such as sterilization, inoculation, incubation, and processing can be carried out in one space without the need for a separate sterilized environment. Although such conditions are typically discouraged by biotechnological companies, which favor compartmentalization to prevent cross‐contamination between stages, targeting large volumetric components and using non‐standard substrates (e.g., bulk wood, pre‐assembled scaffolds, knitted or braided substrates) makes it essential to simplify and minimize transfers between the SSF, particularly for sterilization and inoculation.

An illustrative example is the use of pasteurization as an alternative to autoclaving. Specifically, pasteurization involves the prolonged submersion of the substrate in boiling water. It is preferred over autoclaving from an environmental perspective but also from an operational point of view in case of a lack of availability of large autoclaves. This versatility allows for easy relocation of the fabrication units to onsite locations or existing manufacturing facilities, such as fab labs, robotic automation labs, or timber factories. The potential applications include working with assembled substrates that cannot be pre‐grown or are challenging to handle with standard sterilization and inoculation practices. These applications might involve creating stay‐in‐place formworks, textile membranes, pre‐assembled wooden scaffolds, or woven natural fibers. The degree of sophistication of these controlled environments determines their capability for precise monitoring. This meticulous monitoring, in turn, leads to increased consistency in material properties.

#### 3.3.3 Controlled Environments for Bulk Wood (BULK‐CEn)

**Figure 6:**
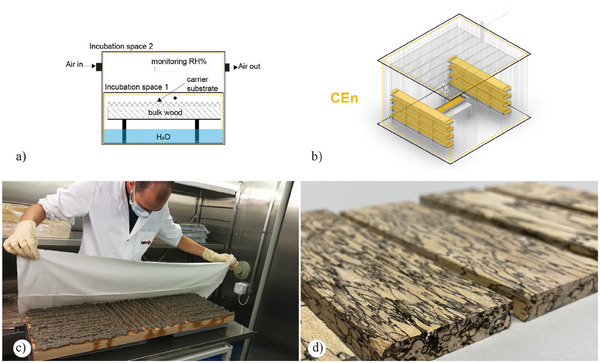
a–d) The conceptual illustration describes the fundamental components of a BULK‐CEn: the dashed line symbolizes the volume of the double controlled environment. a) Sectional diagram of the adaption of the bioreactor and its fundamental components. b) Volumetric drawing of the potential scale‐up and distribution of the operational area. c) Process showing inoculation of wood with a water‐based carries substance and subsequent spalted wood production, Empa Facilities – Sankt‐Gallen, Switzerland. d) Spalted wood results show different zone line patterns emerging from melanin pigmentation. c,d) Reproduced with permission by Empa.^[^ ^129^ ^]^

These environments are designed and developed to manufacture value‐added wood products and primarily treat bulk wood. BULK‐CEn needs to meet the specific requirements of fungal species and the performance of particular value‐added wood products (increasing the value of the wood range). These environments are unique and require extensive scientific research to understand the behavior/biology of the fungal strategy of wood colonization within the manufacturing environment (**Figure** **6**). For instance, fungal species belonging to the *ascomycetes* require special isolated and semi‐sterile conditions for growth. Unlike the typical One‐Factor‐at‐a‐Time (OFAT) approach, these environments necessitate the simultaneous comparison and evaluation of multiple factors.^[^ ^127^ ^]^ Hence, a continuous interaction between a controlled environment and the fungal species is essential for assessing important process parameters and designing experiments. This approach enables the efficient analysis of various combined parameters, such as temperature, substrate water activity, and pH levels. An example of utilizing these environments is the production of spalted wood.

Spalted wood is a specific decay involving wood pigmentation caused by fungal activity.^[^ ^128^ ^]^ Fungal pigment production in the wood substrate is a significant characteristic of spalted wood. Several fungi can produce spalted wood, resulting in the accumulation of black melanin pigment in fine demarcation lines, often accompanied by discoloration or staining of the wood fibers. An example of such a process is the spalted wood project developed by Empa in partnership with Koster AG. The project's objective was to create a simple, rapid, and reliable wood spalting method that can be implemented in the furniture market.^[^ ^26^ ^]^ A chamber filled with H_2_O (note: the wood does not come into direct contact with the water) produces spalted wood. Once the wood is placed in the chamber, it is inoculated with a mycelium/water‐holding carrier substance consisting of nanocellulose and sodium polyacrylate. The environmental conditions exposed to the host wood substrate during the initial colonization and throughout the fungal incubation period significantly influence decay and pigmentation patterns.

#### 3.3.4 Summary

The presented CEns support the production of fungal‐based materials into an established technological framework of prefabrication and modular construction. The design and engineering of such controlled environments enable customization and manufacturing with high complexity and integration into current automation strategies (**Table** **3**). Specifically, INT‐CEns, due to their volumetric capacity, are prone to be adapted to a large spectrum of pre‐fabrication ranges from non‐volumetric, volumetric, and modular building elements and automated processes in sterile conditions. BULK‐CEn, on the other hand, supports the production of non‐volumetric elements based on bulk wood. Working with smaller production volumes allows monitoring and the consequent certification of individual prefabricated elements, adding value to the final product. However, the potential for failure increases as the fabrication process becomes more complex. STAND‐CEns addresses this issue by providing constant monitoring during the incubation phase. The STAND‐Cen is uniquely tailored to pieces with standard dimensions and can be used to produce one single unit per time. Such conditions favor monitoring and ensure consistency during the incubation phase.

**Table 3:** The prefabrication level of fungal‐based materials is associated with the type of construction building components, their volumetric range, and a favorable controlled environment.

Factors	Description	Example	Volume [m^3^]^a)^	Controlled Environment
a)	*Non‐Volumetric* ^b)^	2D wall elements, prefabricated components with no usage space enclosed, e.g., prefabricated fungal‐based materials partition walls, non‐structural external walls /cladding envelope, double ceilings.	1< =	INT‐CEn, STAND‐CEn, BULK‐CEn
b)	*Volumetric*	Comprehending all 3‐D preassembled units enclosing usable space but not part of the building structure, e.g., volumetric prefabricated rooms and integrated slab elements.	1–3	INT‐CEn, STAND‐CEn
c)	*Modular building*	3D preassembled volumetric units form part of or the complete building structure.	>3	INT‐CEn

^a)^ Volumetric range depending on an average of standard construction building components;

^b)^ Term referred to planar sheets of different thickness.

## 4 Challenges and Opportunities

Integrating fungal‐based materials into current practices adopted by the AEC industry presents fundamental challenges, primarily due to the absence of consolidated models for transferring knowledge “from science to a market.” Such deficiencies hinder the seamless integration of fundamental discoveries and basic research into technological advancements and challenges specific to the construction industry. The process involves rigorous evaluation, optimization, and implementation in practical utilization scenarios. The utilization scenarios should consider the complexity and limitations deriving from the material system, scalability, and integration into a clear technological framework brought by prefabrication and modular construction. In this regard, the characterization, engineering, and design of controlled environments play a crucial role in bridging the constraints derived from the material formation and the industrial scale‐up. The existence of such engineered spaces, even if on a pilot level, is crucial for two main reasons. First, it allows the advancement of novel methodologies for process control and designing a controlled environment for an effective industrial scale‐up of fungal‐based materials for the AEC sector. Second, it supports the advancements in the fundamental relationship between fungal species and environmental variables.

In particular, in‐depth characterization and exploration of these controlled environments offer novel insights and raise intriguing questions within applied mycology, off‐site and onsite construction, environmental assessment, and policy‐making.

### 4.1 Applied Mycology

Many challenges concerning the material performance and durability of fungal‐based materials depend on the scientific development of fungal biology and more in‐depth knowledge of the metabolic and enzymatic transformation caused by the fungal species during the partial degradation of organic substrates. In particular, environmental conditions (e.g., temperature, moisture content, gaseous exchange) play a fundamental role in stimulating or suppressing fungal growth. The ideal conditions for fungal growth are often apparent from naturally decayed wood in nature and can be closely mimicked to obtain similar results under artificial conditions. A successful simulation of the environmental conditions will also reduce the risk of contamination during the production of fungal‐based materials. So far, most of the research carried out in academia or adopted by the industry relies on using a limited range of fungal cultures. The cultures come from generations of laboratory cultures grown on synthetic media, affecting the viability of a fungal strain and generalizing results that are different if wild‐type fungal species are used. Through the development and characterization of controlled environments, academic institutions and industry can accurately monitor material growth and gain insights into how specific environmental conditions may affect the metabolic pathways of wood decay fungi.

### 4.2 Off‐Site and On‐Site Construction

Using MBCs in the AEC industry presents significant challenges, primarily stemming from the difficulty of integrating Solid‐State Fermentation (SSF) as a complementary technological framework for construction. These challenges include scalability in the manufacturing process, material handling, consistency in material performance, complex production variables essential for material formation, and the need to access specialized infrastructure. Currently, these limitations restrict the commercialization of fungal‐based materials primarily to non‐volumetric planar prefabricated sheets and the standardization of molding as the dominant manufacturing technique. In this context, prefabrication emerges as a reliable short‐term solution mitigating the risks associated with the SSF process by providing a high degree of quality control over the expected performance and durability of the material. Novel and agile typologies of CENs represent a crucial opportunity to extend the prefabrication scenario to the production of volumetric components and contribute to the adaption of SFF for novel manufacturing processes. One of them is the in situ self‐assembly of fungal‐based continuous structures. There are currently only a few reported examples concerning the potential for growing construction components directly in situ. In situ self‐assembly of fungal‐based materials remains underexplored, highly dependent on fundamental developments associated with the material formation and novel sterilization techniques for high substrate volumes and resistance to contamination. The incubation of fungal‐based materials in situ would allow more flexibility to adapt the material to site‐specific conditions and decrease logistical constraints and the continuity of forces within an architectural component. Implementing an in situ framework would necessitate rethinking the entire building system and design process to adapt to an onsite bioreactor. This condition would require the presence of a high‐performance structural skeleton, identifying areas where materials could be incubated, identifying reliable operational parameters for the expected material performance, and a reduced risk of contamination. Partial achievement could involve isolating/incubating the material by floor or transforming the building's structural skeleton into a continuously engineered, controlled space.

### 4.3 Policymaking

The regulatory landscape around MBCs, is constituted by two main regulatory frameworks. The first refers to MBCs as mushroom‐mycelia products (MMP) and associated food‐derived products, and the second refers to European CE Markings and CPR regulations for construction products. Companies producing construction materials become uncertain when referring to novel food regulations when using agricultural products as suitable substrates for the growth of mycelia. The association of a material to the food industry also makes the certification process more problematic as it requires a material to respond simultaneously to safety assessments from the food industry (nutritional values, toxicology, allergenicity tests, etc.) and the CE marking regulations associated with construction products. Such regulatory conditions also imply that any construction material's expertise is typically optional. It would be necessary to align a new regulatory system to geographical locations and specific local supply chains with a clear legal framework. Pairing a new regulatory framework with CENs and their capacity to monitor and ensure consistency can help increase trust in the material manufacturing and certification process and develop novel regulatory principles that align with the demands and expectations of the AEC industry.

### 4.4 Environmental Assessment

According to a study by Stelzer (2021), producing MBCs on the lab scale shows that using energy‐inefficient technologies leads to high energy intensity, affecting the material's cost and overall carbon profile. Among the most energy‐intensive operations, we find drying ovens, molding preparation, autoclaves for sterilization, and incubation in a controlled environment. When producing MBCs at an industrial scale, it is essential to address the issue of high energy consumption in all steps. For instance, pasteurization of substrates (by immersion at 60 °C for 8 h), reduction of molding, implementation of an efficient waste management system, and utilization of improved monitoring tools can drastically improve the environmental impacts associated with electricity consumption. In this regard, the characterization of controlled environments has twofold implications. On the one hand, offering real‐time control of growth can be used as an aid to current environmental assessment tools. On the other hand, they allow integrative, alternative manufacturing processes to molding and reduce the most energy‐intensive operations.

## 5 Conclusion

Integrating MBCs into the AEC industry presents a promising venue for advancing sustainable and novel circular building practices. However, such a transition comes with a series of challenges. One major obstacle lies in the need for consolidated models for effectively transferring scientific research into practical applications. Furthermore, the demand for specialized resources and infrastructure, coupled with the intricate interplay of biological, physical, and environmental variables during manufacturing, presents considerable obstacles to seamless industrial integration. To address these challenges, leveraging well‐established industrial processes from diverse sectors, such as those employed in mushroom farms, emerges as a promising resource for applying CEns in the AEC sector. This approach offers a practical solution by drawing on the success and adaptability of existing practices, streamlining the incorporation of CEns into the complex dynamics of the AEC industry. Controlled environments provide optimal conditions for fungal growth, ensuring optimal material performance and scalability while affecting the consequent material cost, energy profile, and legal framework of material certification. This article emphasizes how understanding and characterizing such controlled environments plays a role in diversifying manufacturing methodologies, enabling innovative prefabrication strategies, and expanding the range of applications for MBCs.

The successful integration of MBCs into the AEC industry may serve as a blueprint for departing from conventional extraction‐based manufacturing methods to novel circular models that harness the valorization of biological resources. For instance, this idea can be demonstrated by circular bio refineries connected to producing materials, which use energy generation, waste stream exploitation, and industrial symbiosis to make high‐value bio‐based products. Circular biorefineries convert organic waste materials into premium construction supplies using microbial and fungal processes. The amalgamation of material production, waste management, and energy and food production creates a web of interrelated procedures that promote novel approaches to prefabrication and inventive uses of materials derived from fungi.

## Conflict of Interest

The authors declare no conflict of interest.

## Author Contributions

T.D. performed conceptualization; T.D., B.D, and F.S. did an investigation; T.D. prepared and wrote the original draft; T.D., F.S, and B.D. wrote, reviewed, and edited the final manuscript; T.D. performed visualization; F.S, B.D. supervised the project; T.D. performed project administration; B.D. acquired funding. All authors have read and agreed to the published version of the manuscript.
